# Intra-operative Ultrasound-guided Thrombectomy and Thrombolysis for Post-operative Portal Vein Thrombosis in Living Liver Donors

**Published:** 2015-02-01

**Authors:** O. Abdelaziz, K. Hosny, O. Elmalt, S. Emad-Eldin, A. Hosny

**Affiliations:** 1 Liver transplantation team, Dar Al-Foad hospital, Cairo, Egypt; 2 Department of Diagnostic and Interventional Radiology, Cairo University Teaching Hospitals, (Kasr Al-Ainy), Cairo, Egypt; 3 Department of Surgery, Cairo University Teaching Hospitals (Kasr Al-Ainy), Cairo, Egypt; 4 *Department of Surgery, National Cancer Institute, Cairo University, Cairo, Egypt*

**Keywords:** Living donor, Liver transplant, Postoperative complications, Venoplasty, Thrombectomy, Portal vein, Thrombolytic therapy

## Abstract

There are few reports of portal vein thrombosis among living donor liver transplant donors and no published data on the management of this event. In this report, we present our experience in the diagnosis and management of this rare complication in two living donor liver transplantation donors who developed post-operative portal vein thrombosis. Both cases were successfully managed with intra-operative ultrasound-guided thrombectomy, vein patch venoplasty, and catheter-directed thrombolysis. The two donors are symptom-free two years after the event.

## INTRODUCTION

Living donor liver transplantation (LDLT) is the only life-saving cure for end-stage liver disease as well as hepatocellular carcinoma in countries where deceased donor liver transplantation is not permitted, as in Egypt. There are many reported complications in liver donors. Amongst the devastating life-threatening complications is post-operative donor portal vein thrombosis (PVT) reported in only few series [1-4]. The exact incidence and causes are unknown.

Predisposing factors for PVT include hereditary and acquired hypercoagulable disorders, technical factors during hepatectomy, release of thromboplastin and serotonin from damaged tissues and platelets, the increase of pro-coagulant factors induced by an interleukin-mediated (interleukins 6 and 8) acute phase response, and the reduction of anticoagulant factors determined by increased consumption [5-8]. 

Furthermore, removal of a considerable hepatic mass results in diminished hepatic synthesis of anticoagulants [7]. More than half of the healthy liver donors rapidly develop a hypercoagulable state following right hepatectomy, despite the use of prophylactic anticoagulation [8].

PVT occurs mainly in the immediate post-operative period. It may present with liver failure, splenomegaly, ascites, and gastrointestinal bleeding secondary to esophageal variceal rupture [9]. It can be fatal if diagnosis and treatment are delayed [1, 10].

Although reports about this complication have already been published, no previous management details have been reported. Herein, we report our experience in the diagnosis and management of post-operative PVT in two living liver donors. 

## CASE REPORTS

480 LDLTs had been performed at Dar Al-Fouad Hospital and Sahel Teaching Hospital in Egypt between August 2001 and May 2014. There were two (0.42%) cases of donor PVT amongst these 480 donors. The two donors are symptom-free two years after the event.

Case 1

The first donor was a 28-year-old man who came to donate to his brother who had hepatitis C-related liver cirrhosis. His blood group was A^+^. He had a weight of 66 kg, height of 182 cm (BMI of 20 kg/m^2^), no previous medical conditions, normal physical examination, no laboratory abnormalities, and took no medications (Table 1) 

**Table 1:** Laboratory findings of the two patients

Parameter	Case 1	Case 2
Hb (g/dL)	13.9	16.0
Hematocrit (%)	43.0	45.7
Platelets (10^3^/µL)	277	185
Prothrombin time (s)	12	14
Prothrombin concentration (%)	100.0	89.0
INR	1	1.1
Activated partial thromboplastin time (aPTT) (s)	27.0	29.9
Protein S, protein C, antithrombin III	Normal	Normal
Antinuclear antibody (ANA) (by ELISA)	Normal	Normal
Anti DNA (ds)	Normal	Normal
Liver kidney microsomal antibody (LKMA)	Negative	Negative
Anti smooth muscle antibody (ASMA)	Negative	Negative
Factor V Leiden	No mutation	No mutation

The patient donated a right lobe liver graft without the middle hepatic vein weighing 850 g. The pre-operative total liver volume measured by CT volumetry was estimated at 1400 g, which left the donor with a residual left lobe of 550 g (39%). The estimated blood loss was 250 mL; no blood transfusion was needed. The operative time was 8 hrs 45 min, which was longer than our average 6 hrs 30 min, due to a delay in the recipient hepatectomy (Table 2).

**Table 2:** Operative data in the two patients

Parameter	Case 1	Case 2
Graft weight (g)	850	700
Total liver volume (g)	1400	1100
Remnant liver weight	550 g (39%)	400 g (36%)
Unit of transfused blood	None	None
Operative time	8 hrs	7 hrs, 20 min

Adequate hydration of the donor was ensured with fluid balances of +1500 mL/24 hrs, +1350 mL/24 hrs, +95 mL/24 hrs, and +2591 mL/24 hrs in the first four days, followed by a net balance of around 0 mL/24 hrs on the subsequent days. The central venous pressure (CVP) was kept above 5 mm Hg; the urine output was satisfactory. 

**Figure 1:**
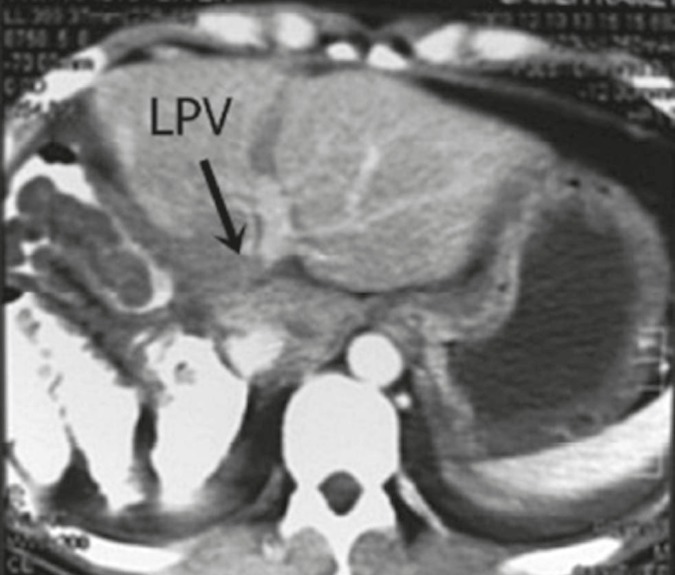
Triphasic CT portal phase showing a thrombus in the main and left portal vein (arrow) and opacified segmental branches.

On the fourth post-operative day, thrombosis of the portal vein was detected by routine duplex ultrasonography and confirmed by CT (Fig 1). The liver enzymes were not elevated but the lactate was mildly high. Surgical exploration was done. Intra-operative ultrasonography (IOUS) was done using a sterilized T-shaped linear probe with a central frequency of 7.5–10 MHz, which showed the presence of clots. A small transverse 3-mm venotomy was done in the main portal vein, 1 cm proximal to the bifurcation on the accessible right aspect of the vein, since the common bile duct and the left hepatic artery are anterior to the vein. The clot was fresh and soft, so a size 7 Fogarty balloon catheter allowed extraction of the liquefied clots, which was aided by digital milking of the loose thrombus out of the vein. The Fogarty catheter was passed several times until no more thrombus was obtained. When the catheter was passed proximally an atraumatic vascular clamp was placed distal to the venotomy so the clots were directed out of the vein instead of into the liver, and when the Fogarty was passed into the liver a clamp was placed proximal to the venotomy. The usual practice is to deflate the balloon when it approaches the venotomy to avoid tearing the venotomy and to milk the remaining thrombus digitally. This was followed by a gush of blood from the proximal inflow as well as the distal back bleeding from the liver. The venotomy was oversewn with 5-0 interrupted polypropylene. 

**Figure 2:**
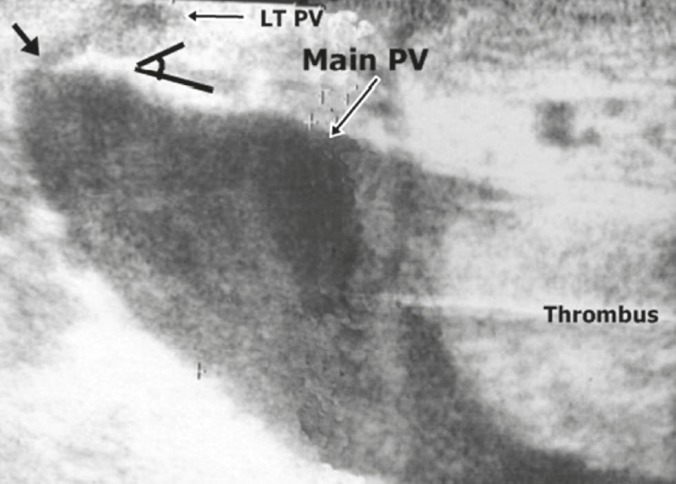
IOUS image showing sluggish flow in the portal vein by B mode, stenosis at the origin of the left hepatic vein (short arrow) and residual proximal thrombus in the portal vein extending to the superior mesenteric and splenic veins.

**Figure 3:**
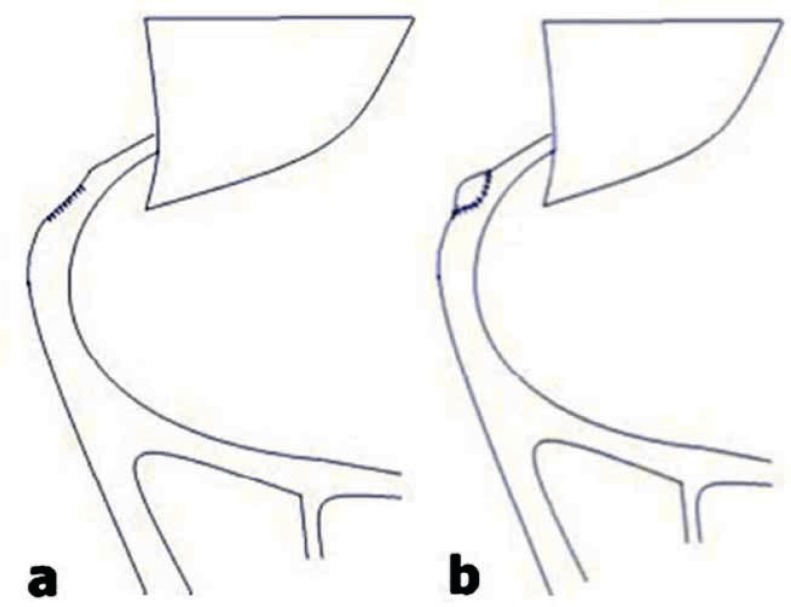
Drawing showing the vein patch at the origin of the left portal vein

**Figure 4:**
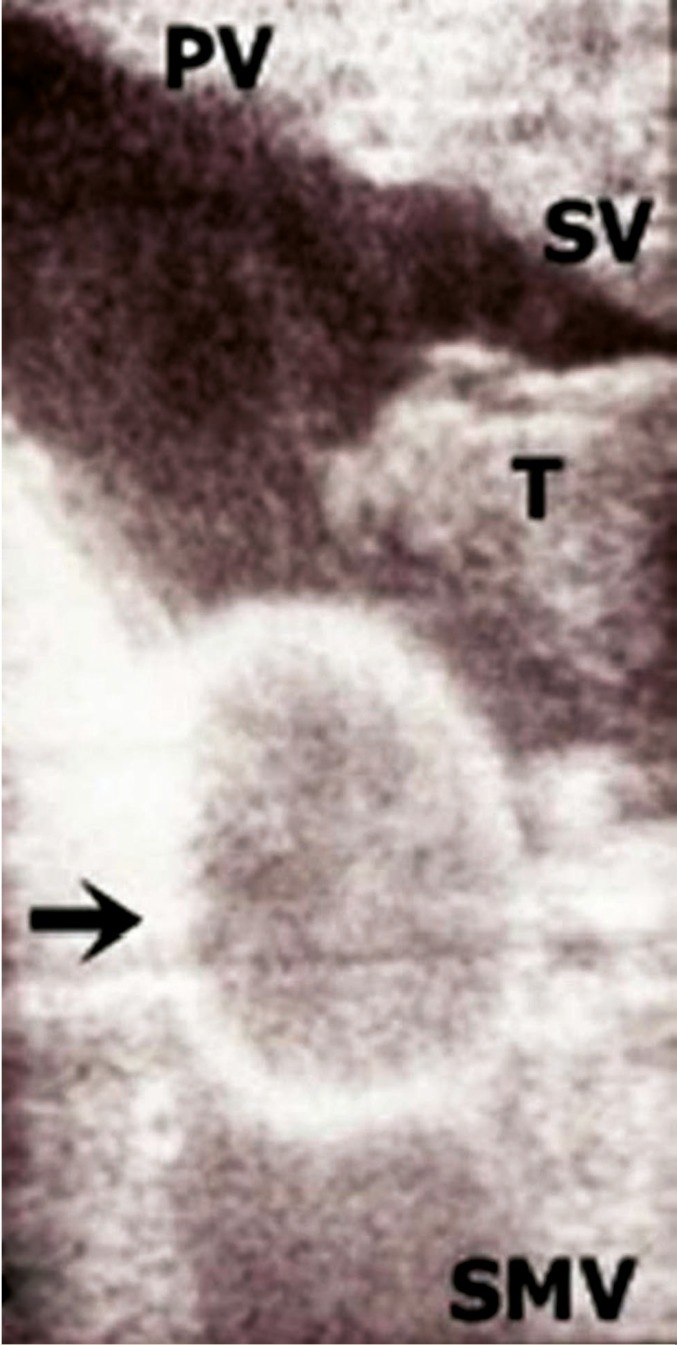
IOUS images showing the balloon of Foley catheter (black arrow) in superior mesenteric vein with residual thrombus in the splenic vein.

At this point the IOUS showed restoration of flow to the liver. However, there were two problems: The first was stenosis at the origin of the left portal vein and acute angle between the main and left portal vein from torsion of the remaining left lobe. The second was a remaining thrombus at the confluence of the superior mesenteric vein and splenic vein (Fig 2). The first step was to correct the stenosis by placing a venous patch from great saphenous vein. A small double angled partial side occluding atraumatic vascular clamp (Satiniski) was placed on the oversewn right portal vein stump. This allowed the liver to be perfused while placing the patch. The 5-0 continuous polypropylene sutures from the initial right donor hepatectomy surgery was cut and the currently opened right portal vein ostium was closed with an elliptical patch using a valveless reversed saphenous vein segment (Fig 3). After removal of the clamp, the angle between the main and left portal vein was less acute and of a larger diameter. The falciform ligament was reattached to avoid kinks. Attention was then directed to extracting the residual thrombus, making sure the catheter was passing into both superior mesenteric vein and splenic vein under ultrasonography guidance. We used a size 16 French Foley catheter for the thrombectomy via the initial 3-mm venotomy and inflated it with 3 mL of saline to avoid rupture the vein from over inflation (Fig. 4), using the same thrombectomy principles mentioned above. Heparin infusion was started intra-operatively and continued post-operatively to maintain a PTT 70–90 sec. Seven units (250 mL) of packed red cells were given. Four units were given intra-operatively to replace the 1000 mL bleeding and three units were administered over 24 hrs to replace the continuous oozing from the incision. 

**Figure 5:**
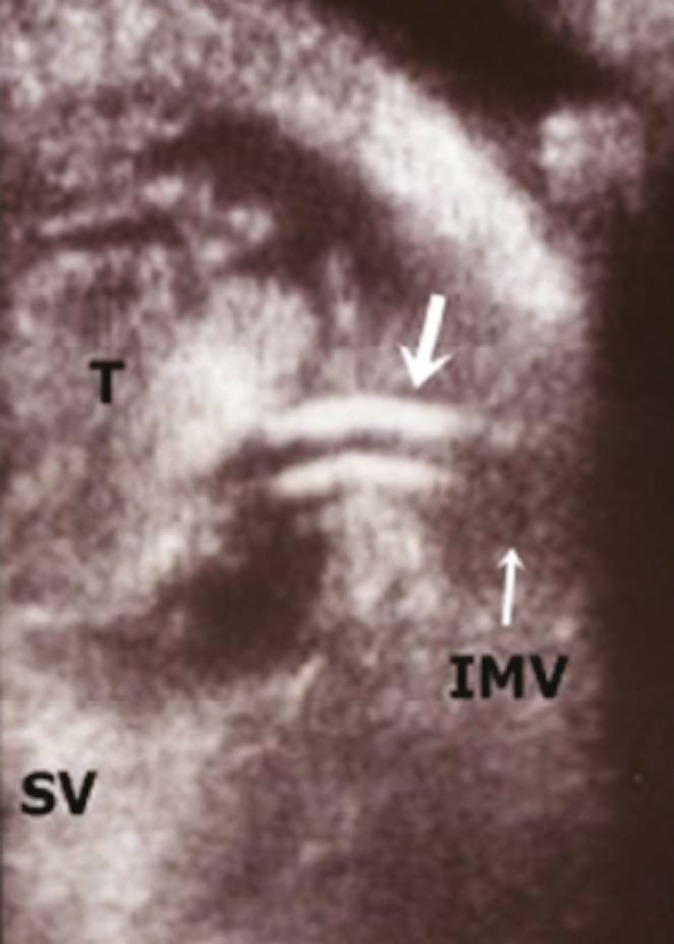
IOUS image: axial section in the splenic vein showing residual thrombus and the tip of the inferior mesenteric vein catheter in thrombus (arrows)

On the fifth post-operative day re-thrombosis of the portal vein was detected again. The enzymes were declining. A second surgical exploration was done. IOUS showed diffused partial thrombosis of the segmental branches of the left portal vein. Portal vein thrombectomy was done again via the same venotomy. However, we used a smaller Fogarty catheter (size 4 and 3) into the liver. IOUS showed restoration of blood flow. However, a residual thrombus in the splenic vein was detected. Therefore, we decided to insert a thrombolysis catheter into the inferior mesenteric vein. We used a Fountain^®^ Infusion System (Merit Medical Systems, Inc) catheter size 4 French, 90 cm long, with a 20-cm side holes segment inserted over a 0.035” soft wire. Under ultrasonography guidance the catheter tip was advanced to the main portal vein while the side holes segment was centered in the thrombus. The other end of the catheter was brought out of the abdominal wall (Fig 5). Streptokinase bolus of 250,000 units was given over 30 min via the inferior mesenteric vein catheter, after which the IOUS demonstrated a patent portal vein and its segmental branches. Streptokinase infusion was continued at a rate of 100,000 U/hr for 24 hrs via the inferior mesenteric vein catheter. The patient continued on heparin infusion as well and a target activated clotting time of 200–300 was achieved. Four units of packed red cells were given over the following 48 hrs to replace the oozing.

On the seventh post-operative day, the inferior mesenteric vein catheter was removed after confirmation of the patency of portal vein and intrahepatic branches by duplex sonography. Warfarin was started and titrated to a target INR of 2–3 and continued for six months. The patient recovered gradually, and was discharged on post-operative day nine in stable condition with no complaints or laboratory abnormalities. 

During the follow-up visits, the laboratory values were normal and duplex sonography showed patent main and left portal vein with minimal partial chronic occlusion of the intrahepatic segment 2 portal vein. The patient remained asymptomatic after two years of follow up. 

**Case 2**

The second donor was a 21-year-old man who came to donate to his uncle who had hepatitis C-related liver cirrhosis. His blood group was O^+^. He had a weight of 64 kg, a height of 166 cm (BMI of 23 kg/m^2^), no previous medical conditions, no history of using medications, normal physical examination, and no laboratory abnormalities (Table 1).

The patient donated a right lobe liver graft without the middle hepatic vein weighing 700 g. The pre-operative total liver volume estimated by CT volumetry was 1100 g, which left the donor with a residual left lobe of 400 g (36%). The estimated blood loss was 200 mL; no blood transfusion was needed. The operative time was 7 hrs 20 min, which was longer than our average of 6 hrs 30 min (Table 2). 

Post-operatively, adequate hydration of the patient was ensured; the CVP was kept above 5 mm Hg; the urine output was satisfactory. 

On the fourth post-operative day, PVT was detected by routine duplex sonography that showed no flow in the main and left portal vein, which was confirmed by CT portography. The liver enzymes were declining at the time of diagnosis, with no acidosis. Surgical exploration and IOUS-guided portal vein thrombectomy were done, in a similar way the first case was managed, using a balloon catheter through a venotomy in the main portal vein. A vein patch was sewn to the right portal vein stump, as in the first case. At the same time, an inferior mesenteric vein catheter was placed under ultrasonography guidance and brought out of the abdominal wall. A streptokinase bolus of 250,000 units was given over 30 min via the inferior mesenteric vein catheter; then, infusion was continued at 100,000 U/hr monitored by sonography every 4 hrs. The infusion was stopped after 12 hrs when doppler ultrasonography showed the patency of the portal vein. Heparin infusion was started to maintain a PTT of 70–90 sec. Eight units of packed red cells were given: four during the thrombectomy to replace 100 mL bleeding and four over the following 24 hrs. 

On the fifth post-operative day, the patient was re-explored to evacuate an intraperitoneal hematoma and to remove the inferior mesenteric vein catheter. IOUS showed adequate flow in the portal vein and intrahepatic branches. The patient continued on heparin infusion and a target activated clotting time of 200–300 was achieved. Warfarin was started and titrated to attain a target INR of 2–3. 

During follow-up visits, the laboratory values were normal and duplex ultrasonography showed patent main and left portal vein. The patient was asymptomatic after two years of follow up.

## DISCUSSION

We found two donors who developed post-operative PVT among 480 living liver donors, which translates to a rate of 0.41%. Other transplant groups have reported the rate between 0.3% and 0.5 % [1-4]. Our two patients experienced the disease during early phase of our LDLT experience. Moreover, the experience learned from the first case made the management of the second one much easier. 

Donors for LDLT undergo an extensive workup prior to donation to assess their suitability and safety for donation. Workup includes identifying hypercoagulable states that contraindicate donation. Our two donors had no past or family history of thrombotic events, with normal laboratory workup. At our institution, we do not perform other lab tests associated with thrombosis as homocysteine, cryoglobulins in serum, ceruloplasmin level, lupus antibodies, anticardiolipin antibodies, antiphospholipid syndrome, prothrombin (factor II) gene mutation or dysfibrinogenemia. Analysis of platelet function can show evidence of accelerated platelet clumping related to increased von Willebrand factor activity (normal range: 50% to 150%), an abnormality shown to be a predisposing factor for thromboembolic events [11]. We will consider adding some of these labs to the workup in the future. 

**Figure 6:**
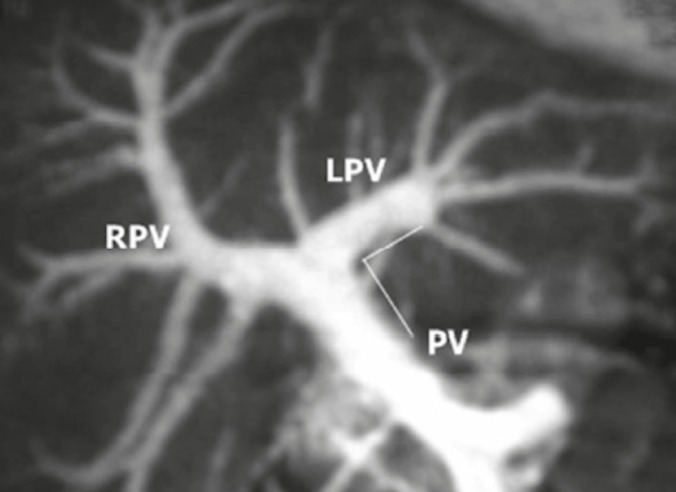
CT portography of Case 2 showing adequate length of the right portal vein and satisfactory angle between the main and left portal veins

Pre-operative portal vein anatomy was studied with CT portography and showed a normally bifurcating main portal vein in the two donors, with an angle of 80° to 85°, which was acceptable (Fig. 6). A short extrahepatic right portal vein may pose a difficulty, with encroachment on the portal bifurcation leading to stenosis. It is know if a more acute angle between the main and left portal vein would induce stasis and thrombosis because of slight torsion of the left lobe.

Intra-operative events associated with thrombosis include technical factors leading to constriction of the vein as cutting the right portal vein too close to the bifurcation, or using stitches that are too far from the vein edge. To avoid this, we used atraumatic vascular clamps, which are placed on the right portal vein a few millimeters away from the portal bifurcation. Inclusion of venous adventitia inside the lumen provides a medium for thrombus formation; therefore, the vein edge should be everted rather than inverted. Meticulous hemostasis is important to prevent hematoma formation, which may compress the portal vein, and cause thrombosis. Long operative time should be avoided; in both donors the operative time was much longer than usual, which may have augmented the hypercoagulable response. During surgery we routinely re-attach the falciform ligament after right hepatectomy prior to closure of the donor abdomen to fix the liver in position to prevent kinking of the liver pedicle.

In both cases, adequate post-operative hydration was ensured with ample fluid replacement and monitoring the CVP, which was kept above 5 mm Hg, and satisfactory urine output above 50 mL/hr.

It was shown in a study by Levitsky, *et al*, that both post-operative prothrombotic and proinflammatory states were strong in living kidney donors, as evidenced by the a decline in activated protein C level during the first post-operative day, which took up to 90 days to return to normal. There are also elevated highly sensitive C-reactive protein, which also took up to 30 days to return to the baseline, as well as increased D-dimer levels [12]. Cerutti and his colleagues demonstrated that donors of the right liver lobe might develop hypercoagulability by thromboelastograms on postoperative days 5 to 10 and even clinical thrombosis. They therefore kept their donors on low molecular weight heparin for 20 days. Administration of activated protein C or antithrombin III could also be considered [8]. Our protocol is to start prophylactic anticoagulation immediately before surgery in the form of the low molecular weight heparin, enoxaprine, 40 mg subcutaneously daily, which is continued for one week until the hypercoagulable state is in remission, although some centers do not routinely administer anticoagulation [13]. 

Routine duplex ultrasonography and a full laboratory profile were performed every 12 hrs after donation. It is important to perform routine surveillance duplex sonography and to have a high index of suspicion to detect PVT as early as possible, because the liver enzymes and lactate may not rise initially. It may even be possible to predict impending thrombosis when bidirectional flow is seen [14]. If thrombosis is detected, we recommend performing an IOUS-guided thrombectomy, inserting a vein patch on the origin of the right portal vein, and inserting an inferior mesenteric vein catheter, all at the same time, during the surgical exploration. The thrombectomy can be done via the right portal vein opening without performing a separate venotomy in the main portal vein, if the decision for patch venoplasty has been taken to save time.

If there is residual intrahepatic thrombus not amenable to thrombectomy, thrombolytic therapy should be started with an intra-operative bolus followed by infusion over 24 hrs. We reported a bleeding complication from the streptokinase dose in the second case, thus we recommend using a smaller dose of streptokinase as a bolus of 100,000 units followed by 50,000–75,000 U/hr or recombinant tissue plasminogen activator (rtPA) instead. 

The value of IOUS is to exactly delineate the extent of the thrombus, to make sure the Fogarty catheter enters both splenic vein and superior mesenteric vein proximally, and into the affected segmental liver portal vein branches distally, to verify correct placement of the thrombolysis catheters, and to verify complete thrombus removal and adequate flow thereafter. Shifting and tilting of the ultrasonography probe allows understanding the 3D spatial relationship, as has been described in recipient thrombectomy [15].

In conclusion, donor PVT is a life-threatening emergency and one of the gravest complications after LDLT. The refinements of the technique in donor hepatectomy should be advocated in every case to emphasize donor safety. Identifying donor hypercoagulable states, early detection of PVT with doppler ultrasonography surveillance and rapid surgical intervention are life-saving. IOUS-guided thrombolysis, thrombectomy and venoplasty may aid to ensure complete thrombus removal and adequate portal flow. 
